# An Approach to Identifying Single-Nucleotide Mutations Using Noncovalent Associates of Gold Nanoparticles with Fluorescently Labeled Oligonucleotides

**DOI:** 10.3390/ijms252413230

**Published:** 2024-12-10

**Authors:** Anna V. Epanchintseva, Ekaterina A. Gorbunova, Mikhail D. Nekrasov, Julia E. Poletaeva, Inna A. Pyshnaya

**Affiliations:** Institute of Chemical Biology and Fundamental Medicine SB RAS, Novosibirsk 630090, Russia; ekaterina@inbox.ru (E.A.G.); unispell@yandex.ru (M.D.N.); fabaceae@yandex.ru (J.E.P.)

**Keywords:** drug-resistant forms of tuberculosis, single-nucleotide polymorphism, fluoroquinolones, gold nanoparticles, tandem duplexes, noncovalent adsorption, colorimetric detection, hydrophobic oligonucleotide derivatives

## Abstract

Globally, widespread tuberculosis is one of the acute problems of healthcare. Drug-resistant forms of tuberculosis require a personalized approach to treatment. Currently, rapid methods for detecting drug resistance of *Mycobacterium tuberculosis* (MTB) to some antituberculosis drugs are often used and involve optical, electrochemical, or PCR-based assays. Despite the large number of these assays, it is necessary to develop new tests (for drug-resistant MTB strains) that are structurally simple and do not require specialized equipment. Colorimetric assays involving a colloidal solution of gold nanoparticles (AuNPs) have good potential for the development of the needed diagnostic tools. Here, conditions were found for the formation of tandem duplexes between DNA probes and DNA targets, representing a part of MTB gene *gyrA*, either wildtype or containing a single-nucleotide polymorphism associated with fluoroquinolone resistance of MTB. Adsorption of the duplexes on AuNPs allowed to distinguish the two targets owing to the formation of nano-constructs of different structures. Interaction of DNA with AuNPs was analyzed by optical spectroscopy, dynamic light scattering, and transmission electron microscopy. A scheme is proposed for direct colorimetric detection of the fluoroquinolone-resistance-associated single-nucleotide polymorphism at a 2 nM concentration in a liquid system based on a shift of AuNPs’ optical absorption maximum.

## 1. Introduction

Across the globe, the widespread occurrence of tuberculosis, which is the second leading cause of death in the world [1], and the spread of drug-resistant tuberculosis are some of the acute problems in the healthcare sector. Drug-resistant forms of tuberculosis require a personalized approach to treatment based on accurate diagnosis of a specific type of drug resistance [2]. Most of the widely used tests can identify resistance only to common first-line antituberculosis drugs [2], thereby leading to inadequate monitoring of other types of drug resistance. This situation often causes the prescription of an unjustified treatment, which can subsequently result in acquired drug resistance to first-line drugs [3]. In brief, a number of published methods for detecting tuberculosis-associated targets based on AuNPs are reviewed here, including a wide range of technological platforms: optical, electrochemical, and immunoferment sensors, and other, less common ones.

Aside from classic culture methods for detecting multidrug-resistant *Mycobacterium tuberculosis* (multidrug-resistant MTB) [1], rapid tests for detection of drug resistance to fluoroquinolones in sputum samples by high-resolution melt curve analysis [4] or for detection of resistance to rifampicin and isoniazid by multi-fluorescence quantitative real-time PCR [5] are also used in clinical practice. Additionally, the SERS (surface-enhanced Raman spectroscopy) technique holds promise for the detection of multidrug-resistant MTB, in particular, in combination with PCR for the discrimination of rifampicin-resistant strains with the help of principal component analysis [6]. It is possible to employ the genome-editing systems CRISPR-Cas12a and CRISPR-Cas13a to detect MTB strains resistant to streptomycin [7] or to fluoroquinolones [8], respectively. These test systems are aimed at identifying the pathogen in a clinical sample or at assessing its resistance to rifampicin, isoniazid, streptomycin, and fluoroquinolones.

One possible type of diagnostic test for target nucleic acids is based on the complementary interaction of the target either with a single probe or with a tandem of oligonucleotide probes [9,10]. Gold nanoparticles (AuNPs) are promising for the immobilization of oligonucleotide probes owing to their suitable spectral properties, biocompatibility, ease of synthesis, the ability of these particles to effectively adsorb nucleic acids and many other organic compounds, and stability during storage [11]. AuNPs are attractive for the creation of colorimetric diagnostic assays because of these particles’ intense color and an easily visually detectable change in the optical absorption maximum after aggregation at high ionic strength. The AuNP-based detection systems described in the literature are mainly intended to identify antigens of MTB, i.e., detect the pathogen itself in clinical samples. Some published techniques are designed to detect mutations in MTB that are associated with its drug resistance.

There are various electrochemical sensors for the detection of MTB-associated targets. Voltammetric/amperometric electrochemical biosensors have been devised that detect MTB antigens [12,13,14], even at 0.1 femtomoles/L [15]. An impedance-based electrochemical biosensor for the detection of 16 kDa heat shock protein—a potential target of tuberculosis diagnostics—has been described [16]. A DNA biosensor for efficient recognition of a unique DNA sequence of MTB IS6110 is described in [17]. The relative simplicity of electrochemical sensor designs is complicated by the necessity of certified electrical equipment for their use.

Furthermore, there are many optical biosensors involving AuNPs for the detection of MTB. Biosensors based on surface plasmon resonance (SPR) or localized SPR are the most advanced label-free optical biosensor technologies currently available [18]. The use of AuNPs [19] significantly improves the shift of a peak of localized SPR, thus significantly enhancing the detection sensitivity. There are reports of approaches for the detection of point mutations associated with drug resistance of MTB (i) by means of probes immobilized on AuNPs and a target immobilized on the surface of a gold-coated SPR sensor, with a potentially achievable limit of detection as low as 6 nM [20], and (ii) by means of rolling circle amplification [21] or allele-specific PCR [22] and probes on the surface of AuNPs. Such an assay has been developed for interferon-γ (at 0.1 nM), which is involved in the diagnosis of latent tuberculosis [23]. Colorimetric optical biosensors combined with other detectors have been designed, too. Loop-mediated isothermal amplification (in combination with probes on AuNPs) has been adapted for rapid and accurate identification of MTB IS6110 at a level of 5 pg [24]. An antigen–antibody interaction assay has been devised for the recognition of MTB antigen Ag85B [25]. Unfortunately, this class of sensors is characterized by both the complexity of the system design and by the necessity of a high-tech infrared spectrometer for their use. A colorimetric system for CFP-10 (culture filtrate protein 10) detection has been presented, which involves two antibodies attached to AuNPs (intended for detection) and magnetic microparticles (used for separation), with a limit of detection of 0.3 pM [26]; however, such assays for a single-nucleotide polymorphism (SNP) have not been reported to date.

Other types of sensors for MTB detection on the basis of AuNPs have been described as well. A quartz crystal microbalance biosensor for MTB detection at a level as low as 5 pg has been reported [27]. Test strips based on an enzyme immunoassay for automatic diagnostics of infection with MTB have been presented, in which antigen Mtb CFP10-ESAT6 serves as a target that binds to antibodies, while AuNPs are utilized for color development (colored bands signifying the type of result) in the test region and control region of the test strip [28]. Giant magnetoresistance, which occurs when the electrical resistance of a material changes in response to an alteration of the surrounding magnetic field, has been applied to detect the ESAT-6 protein (early secreted antigenic target 6 kDa), which is secreted by MTB, in a magnetic biosensor with a pg/mL limit of detection [29].

The review presented here demonstrates the existing lack of methods for rapid and technologically simple diagnosis of mutations in MTB. Despite the abundance of available diagnostic assays both for MTB itself and for its drug-resistant strains, the design complexity and the need to simultaneously have expensive high-tech scientific equipment in the laboratory lead to unfulfilled demand for new tests. The needed tests are, firstly, structurally simple, and secondly, they do not require specialized or expensive equipment, thereby allowing for a rapid, accurate assay for various types of MTB’s drug resistance.

Our experience [30,31,32,33] enables us to obtain nucleic acid carriers based on AuNPs with programmable surface loading of oligonucleotide probes containing “anchor” components for effective noncovalent adsorption on AuNPs. In this work, in a simple liquid model system based on a shift of the optical absorption maximum of AuNPs at different degrees of their aggregation at high ionic strength, a scheme is proposed for direct colorimetric detection of a single-nucleotide polymorphism (SNP) (at a 2 nM concentration) that is associated with drug resistance of MTB. The method presented here allows rapid and technologically simple determination of mutations in MTB without preliminary target amplification or signal-enhancing techniques. Such approaches are in high demand. The underlying principle is differences in efficiency and in the type of adsorption of DNA/DNA mixtures on AuNPs. A detailed analysis is presented regarding noncovalent interactions of the probes, on the one hand, with complementary targets of various lengths and nucleotide sequences, and on the other hand, with AuNPs having strong affinity for DNA molecules of virtually any composition, including (especially strong affinity) for fluorescently labeled ones. The interactions of DNA with AuNPs were examined by optical spectroscopy, dynamic light scattering (DLS), and transmission electron microscopy (TEM).

## 2. Results and Discussion

### 2.1. The System of Model Targets and Probes Investigated in This Work

Tuberculosis with extensive drug resistance involving MTB’s additional resistance to fluoroquinolone is one of the drug-resistant tuberculoses that are most difficult to treat. In the context of resistance to fluoroquinolones, research attention is focused on a genomic region that determines resistance to fluoroquinolones (a quinolone-resistance-determining region) and covers codons 74–113 in MTB gene *gyrA* and codons 461–499 in MTB gene gyrB [34]. For the current study, we chose the region in gyrA. It has been shown that 63% of ofloxacin-resistant MTB strains contain a mutation in codon 94. This genomic region may contain one of several mutations in codon 94, for example, a SNP at position 280 (A > C), leading to the substitution of amino acid residue Asp94 with Ala. From several known mutations, this one was chosen by us because it leads to the strongest signal in two-stage PCR combined with microarrays [35].

**Figure 1:**
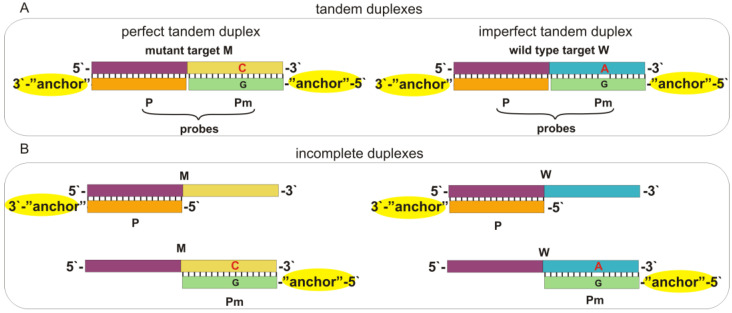
Duplexes formed by probes P and Pm and targets M (mutant) and W (wildtype) for the purpose of detection of the single-nucleotide polymorphism (SNP) in *Mycobacterium tuberculosis* (MTB): tandem duplexes upon cooperative binding/hybridization of both probes to a target (**A**) or incomplete duplexes containing only one probe (**B**). Different colors correspond to different DNA sequences.

We synthesized model systems consisting of targets (34 nucleotides (nt) long) and corresponding probes (17 nt long each). The systems of probes and targets as well as the duplexes they form are depicted in Figure 1.

**Figure 2:**
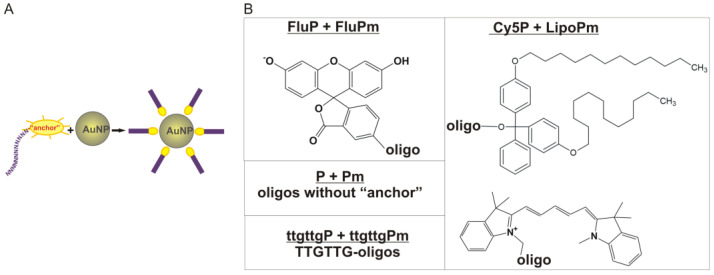
(**A**) The model of adsorption of “anchor” oligonucleotide probes on gold nanoparticles (AuNPs) and (**B**) names of probes and “anchor” structures in the probes.

Probe Pm is fully complementary to the 3′-terminal region of mutant target M containing the substitution in question (a single-nucleotide mismatch: C instead of A) relative to wildtype target W. Probe P is fully complementary to identical 5′-terminal regions of both targets. These oligonucleotides can form: (1) tandem duplexes upon cooperative binding/hybridization of both probes to a target (Figure 1) or (2) incomplete duplexes containing only one probe or a 17 nt overhang. We synthesized four pairs of probes (Figure 2) containing one of three “anchor” types (for efficient adsorption on AuNPs) and differing in hydrophobicity: a fluorescein residue on both probes (FluP + FluPm), a didodecyltrityl group paired with a Cy5 residue at the 3′-end of the second probe (Cy5P + LipoPm), a hexameric ttgttg motif (ttgttgP + ttgttgPm) having strong affinity for AuNPs, and control probes (devoid of any anchor), P + Pm. We chose this motif based on our previous study on obtaining DNA sequences with high affinity to AuNPs using the SELEX (Systematic Evolution of Ligands by EXponential Enrichment) [32]. Later, the positive effect of the inclusion of this hexameric motif TTGTTG in the oligonucleotide on the adsorption efficiency on AuNPs was shown [30].

The use of probes with potentially different rates and strengths of the adsorption of “anchor” regions is a tool for controlling the processes of duplex formation and of adsorption of DNA on AuNPs, and this tool helps to find optimal parameters for detecting two targets [30].

Because it is known that the best discrimination of nucleotide mismatches in a duplex can be achieved at the melting temperature (Tm) of the duplex [36], we investigated thermodynamic stability of the duplexes formed by each target and probe at different DNA concentrations and ionic strengths of solutions, namely, in (1) 10 × 10^−6^ M DNA solutions in the presence of 5 mM phosphate-buffered saline (PBS) or in (2) 0.3 × 10^−6^ M DNA solutions in the presence of 0.3 mM PBS. These are conditions 1 and 2, respectively. The measured T_m_ values for duplexes with Flu- and ttgttg-containing probes are given in Table S1. Condition 1 is optimal for determining the melting temperature values of nucleic acid duplexes by thermal denaturation of DNA solutions with optical signal recording. This concentration condition allows us to identify the possibility of detecting this specific mutation by a method. Condition 2 is the minimum technically possible in the DNA concentration (0.3 μM) for reliable registration of optical density by the selected method. It allows us to estimate how much the Tm values and the difference between them for two targets decrease when DNA concentrations are reduced by two orders of magnitude.

Let us first consider the duplexes formed by pairs of each probe and target under conditions 1 and 2. For probe FluP, which forms a perfect duplex with the invariable part of both targets, higher T_m_ values (almost equal for the two targets) were obtained via measurements, as compared to probe FluPm, in good agreement with the GC content of the corresponding duplexes. The difference in T_m_ between probe FluPm and each of the two targets reached 5.6 °C under condition 1 and 1.6 °C under condition 2. In the analysis of tandem duplexes, two T_m_ values were determined that represent separate melting of each of the two parts constituting the tandem duplex. Presumably, higher T_m_ values (marked by superscripted II in Table S1) correspond to the melting of probe FluP within a duplex, and lower values (marked by superscripted I in Table S1) denote the melting of probe FluPm within a duplex, similar to the Tm values obtained by measurement under condition 1 for pairs of each probe and each target.

Judging by the optical properties of AuNPs and the concentration of the detection target (after amplification of genomic DNA of MTB from a biological sample, the number of amplicon molecules will be ~1011 [37]), the assay should have sensitivity in the nanomolar range of DNA concentrations. At the moment, it is technologically impossible to determine Tm values experimentally at such DNA concentrations and, therefore, we calculated Tm values of duplexes under “condition 3”: DNA concentration = 3 nM in 3 mM PBS using the OligoCalc software v.3.27 in the Salt-Adjusted mode [38] (Table S1). Condition 3 is the real condition in the developed SNP detection protocol and corresponds to the expected target content at the nanomolar level in clinical samples. Experimental data under condition 2 and the calculated data under condition 3 indicated that it is possible to create conditions for more efficient formation of duplexes between probe Pm and target M, as compared to duplexes between probe Pm and target W. This difference can provide a basis for detecting a SNP, similar to [39]. Furthermore, the difference increased from 1.6 to 4.2 °C when we analyzed the melting of tandem duplexes of probe FluPm with targets M and W, implying a cooperative nature of the probe binding within the tandem duplexes (Figure S1A,B). This observation is consistent with findings about a selectivity function studied by thermal denaturation of allele-specific duplexes in [36], which shows that probes have a higher discriminatory ability when used at lower concentrations [40,41]. Thus, we expected effective discrimination of the targets under condition 3, which was tentatively assumed to be suitable for the required limit of detection of the target.

**Figure 3:**
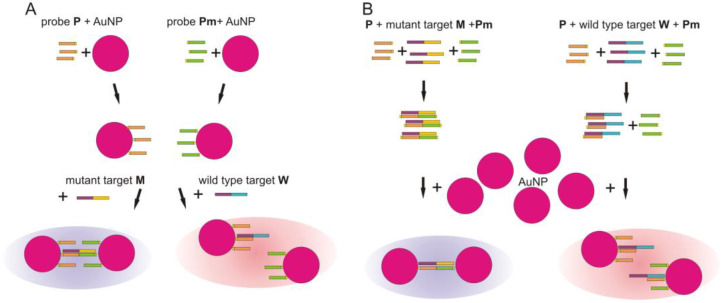
Schemes of the experiment with detection of the SNP. (**A**) Obtaining separately the associates of AuNPs with each probe, P or Pm, at the first stage and then addition of one of the two targets, M (mutant) and W (wildtype). (**B**) Formation of duplexes of the target, M or W, with both probes, P and Pm, at the first stage, then addition of AuNPs, resulting in differences in adsorption efficiency on AuNPs among the formed duplexes. Implementation of any of these detection schemes should create differences in the resistance of the resultant AuNP–DNA associates to salt-induced aggregation. Different colors correspond to different DNA sequences.

Two fundamental schemes for detecting a SNP using AuNPs are theoretically possible and are described in the literature [20,42,43,44] (Figure 3).

The first one (Figure 3A) involved (i) obtaining separate associates of AuNPs with each probe and then (ii) addition of one of the two targets at a certain ionic strength that promotes target discrimination. The second scheme (Figure 3B) involved (i) formation of tandem duplexes of the target with both probes in solution and then (ii) addition of AuNPs to the system. The latter action initiated adsorption of the formed duplexes, of free probes, and of targets onto the surface of AuNPs. In this context, the adsorption effectiveness differed among the three processes, thereby leading to different levels of resistance of the resultant AuNP–DNA associates to salt-induced aggregation [45]. When NaCl solution was added into the AuNP solution, a peak height change at 520 nm was clearly observed, and the plasmon peak shifted toward 620 nm. This phenomenon indicated that the dispersed GNP lost its stability and aggregated as the monovalent ion concentration increased [46,47,48]. The aggregation of AuNP against monovalent ion was investigated considering the hybridization of the DNA probe and DNA sequence with a single base mismatch (wildtype). The disruption of the DNA duplex structure occurred due to the base pair mismatch, which allowed the ssDNA probe and wildtype strand to float freely in its colloidal suspension. Considering the high binding affinity between ssDNA and AuNP, the free-floating DNA strands of both the probe and wildtype strands were attached to the AuNP surfaces. The stable DNA double-helix structure had high electrostatic force in contrast to the bond between ssDNA and AuNP. Thus, at a high concentration of salt solution, AuNP tended to gradually lose its stability as the concentration of mutant DNA increased and AuNP aggregation increased.

### 2.2. Investigation of the Conditions for Mutant and Wildtype Targets’ Discrimination

We implemented several versions of the experimental protocol for detecting the single-nucleotide mismatch. These procedures were designed on the basis of literature data [20,42,43,44,49], and we experimentally tested both of the schemes described above (Figure 3). Each protocol was performed as at least three independent experiments, and each experiment involved four similar samples (prepared independently in an identical manner), analyzed simultaneously. To evaluate the detection results, we employed visual inspection (of photos of samples in test tubes), optical absorption spectroscopy with calculation of the ratio of optical density at 520 nm (i.e., D_520_: maximum optical absorption of the original AuNPs) to D_650_ (the most probable and widely used absorption maximum of aggregated AuNPs: a broad absorption peak in the range of 600–700 nm) [50,51], as well as DLS and TEM.

The first protocol revealed substantial competition between the processes of adsorption of DNA on AuNPs and duplex formation in the system under study (Figure 3A). The efficiency of duplex formation decreased with the addition of AuNPs owing to the adsorption of individual DNA strands on the AuNP surface [30].

**Figure 4:**
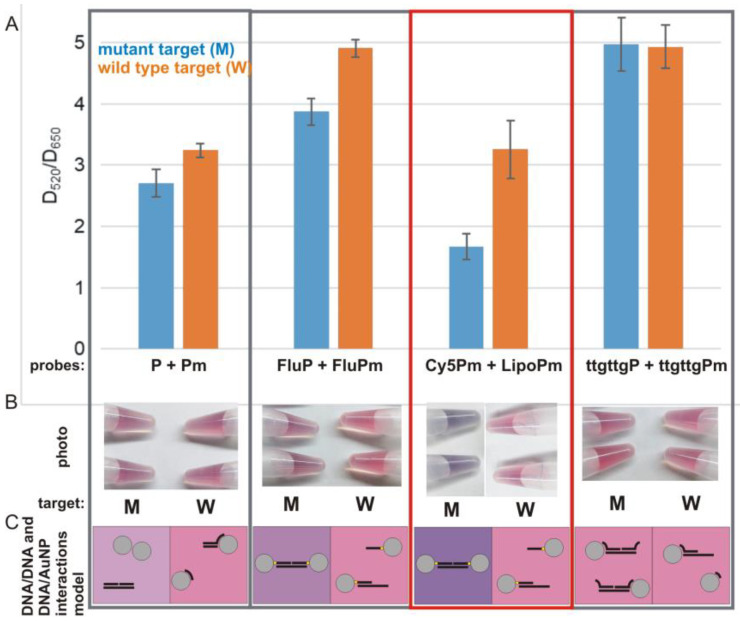
Different levels of differentiation between the mutant target (M) and wildtype target (W) by means of probes P and Pm, carrying different anchors or devoid of them. A histogram of optical density at 520 nm to optical density at 650 nm (D_520_/D_650_) ratios (**A**), photos of samples in test tubes (**B**), and a schematic representation of the adsorption processes and of the formation of duplexes of various compositions in samples containing two different targets (**C**). The background color matches the color of real samples, thereby emphasizing the feasibility of their visual discrimination.

A detailed analysis of T_m_ values at various ionic strengths and concentrations of components prompted us to focus on the second protocol (Figure 3B). It was evident that the use of probes carrying a fluorescein residue enabled reliable visual and spectrophotometric discrimination between the wildtype target and mutant target (Table 1 and Figure 4).

The fluorescent “anchor” promoted rapid adsorption [31] of the tandem duplexes (consisting of the mutant target and two probes) that formed under our detection conditions on the surface of adjacent AuNPs. There was formation of AuNP aggregates, giving a bluer color to the solution (Figure 4). In the sample containing the wildtype target, only associates of stand-alone AuNPs with the noncomplementary probe most likely formed in the solution, as did associates of stand-alone AuNPs with duplexes of the wildtype target and complementary probe. These events resulted in a redder color of the solution. 

**Figure 5:**
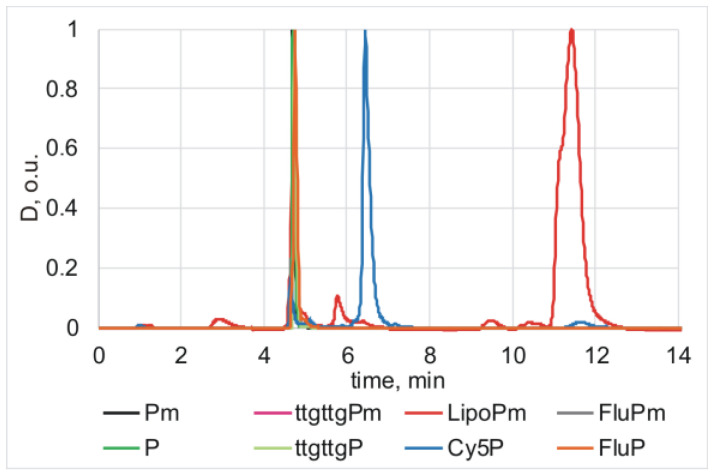
Reverse-phase chromatography of eight probes tested in this work. The maximal optical density (D) of each oligonucleotide is normalized to 1 optical unit.

The use of more hydrophobic anchors in the probes (namely, the combination of the didodecyltrityl group at the 5′-end of probe Pm and the Cy5 residue at the 3′-end of probe P) caused more pronounced aggregation in samples containing the mutant target. This is because adsorption of these tandem duplexes was more efficient, as compared to other types of anchors and to the wildtype target (Figure 4). The hydrophobicity of the probes was assessed via a comparison of the retention times among the oligonucleotides on a column during elution with an acetonitrile gradient. One can see that the introduction of the didodecyltrityl group into probe Pm significantly increased the retention time, from 4.67 to 11.41 min, and the introduction of the Cy5 residue into double probe P also extended the retention time, to 6.43 min (Figure 5).

This observation helped us to further enhance the visual and spectroscopic difference between the samples containing different targets (Figure 4), consistent with our previous finding that a molecule’s hydrophobicity is the leading factor among the noncovalent forces acting during the adsorption of DNA on AuNPs [31]. Our DLS data confirmed the formation of associates having different diameters in samples containing different targets (Table 2).

**Figure 6:**
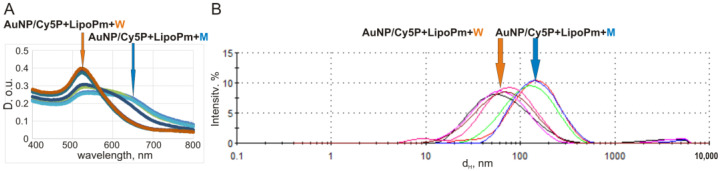
Optical absorption spectroscopy (**A**) and the size distribution of AuNP–DNA associates in samples featuring the didodecyltrityl anchor in probe Pm and the Cy5 residue in probe P and two different targets, M (mutant) or W (wildtype), as determined by DLS in the light scattering intensity analysis mode (**B**). Different lines represent four similar samples (prepared independently in an identical manner).

The hydrodynamic diameter differed by almost two-fold between AuNPs’ associates with target M and those with target W (Figure 6A). This observation theoretically fully matched the formation of AuNP aggregates. Figure 6B clearly illustrates the spectroscopic difference between the two types of samples.

**Figure 7:**
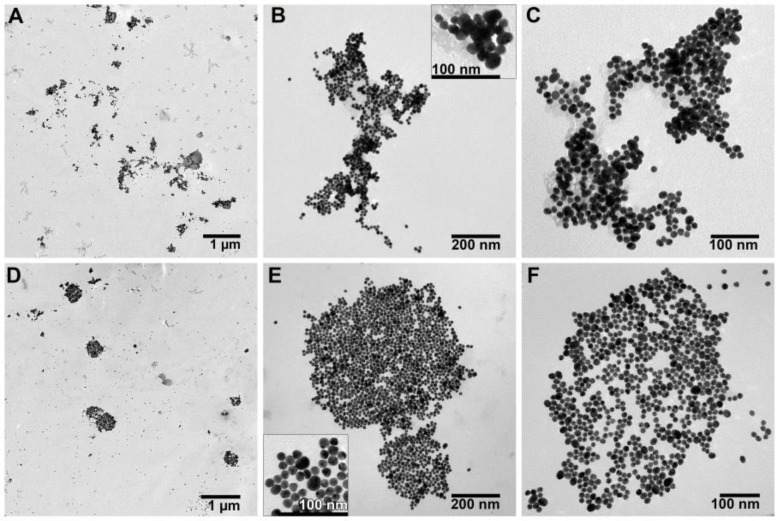
TEM (transmission electron microscopy) micrographs of samples featuring the didodecyltrityl anchor in probe Pm and the Cy5 residue in probe P and two different targets: mutant (M) (**A**–**C**) or wildtype (W) (**D**–**F**). The scale bars are 1 μm (**A**,**D**), 200 nm (**B**,**E**), and 100 nm (**C**,**F**).

Statistical analysis of the Au core diameter was performed based on TEM images. Associates of AuNPs with probes and with target M or W had a spherical shape and an Au core diameter of 14.6 ± 1.9 nm for samples with the M target and 14.6 ± 2.4 nm for samples with the W target (Figure 7), which corresponded to the size of the initial citrate-generated AuNPs (14.4 ± 1.9 nm), so no statistically significant differences were found between the initial Au cores and Au cores modified with targets (*p*-value > 0.05). AuNPs/M/probes were arranged on the supporting film mainly in irregularly shaped clusters, which were single-layered or multi-layered in some regions. Sizes of clusters varied from 290 to 860 nm for samples with the W target and from 30 to 750 nm for samples with the M target. Single particles and clusters of several particles were present, too (Figure 7A–C).

Unlike AuNPs/M/probes, AuNPs/W/probes were organized into rounded single-layer clusters with diameters of 200–600 nm. The particles in clusters were located close to each other or organized into chains, forming dense or loose clusters, respectively (Figure 7D–F). At the same time, AuNPs/W/probes were separated from each other by a clearly visible space of 1.5 ± 0.3 nm. Besides clusters, numerous individual particles and clusters of several particles were uniformly distributed on the grid.

In the probes carrying hexanucleotide “anchor” motifs, one could expect higher stability of the resultant duplexes owing to the lengthening of the probes. Nonetheless, the two types of targets failed to be discriminated—both types of samples had the same red color. It is known that the thermodynamic stability of duplexes increases after introduction of short single-nucleotide overhangs at the 3′- or 5′-end of a probe [52], and further elongation of the overhang may destabilize the duplex [53]. This notion was confirmed by a decrease of experimental and calculated T_m_ values by 1.2–3.1 °C for similar duplexes containing Flu and ttgttg probes (Figure S1C,D and Table S1). In the presented salt concentrations, this finding pointed to the formation of a set of associates of stand-alone AuNPs with probes or target–probe duplexes via multi-contact adsorption of high-affinity anchors [30].

### 2.3. Discrimination Between the Targets Under Control Conditions

The presented scheme of differentiation between the targets (Figure 4C) can be proven by a study on the adsorption of the same sets of probes and targets under conditions that ensure the assembly of tandem duplexes for both the mutant target and the wildtype target. We performed all adsorption steps at 25 °C after 95 °C annealing of probes and targets separately for three pairs of probes, without changing the duration of the adsorption steps (Table 3). No significant difference in the D_520_/D_650_ ratio between the two targets was observed in any probe pairs. All probes retained high colloidal stability and a red color, indicating that aggregates of AuNPs did not arise under the conditions conducive to the formation of tandem duplexes for both target M and target W. Considering (1) the low efficiency of adsorption of double-stranded DNA on AuNPs in general [54], (2) the relation between the rate of adsorption of any DNAs on AuNPs and the incubation temperature [55,56], and (3) the incubation duration in the protocol (~40 min), it can be theorized that there was adsorption both of formed tandem duplexes (a minor fraction) and of free probes and targets (a major fraction) on AuNPs, to a comparable extent when the two targets were compared. As a consequence, we obtained red samples that were stable at high ionic strength.

At the same time, it should be noted that the samples containing fluorescent probes showed a small but significant decrease in the D_520_/D_650_ ratio, indicating the formation of a small proportion of aggregates owing to higher-affinity anchors, as compared to the other two probe types. This outcome confirmed that temperature control is crucial for target discrimination.

Examination of a system containing anchor-free probes should clarify the processes occurring in the system. Anchors in the probes are needed to facilitate AuNP aggregate formation after contact of the formed duplexes (including tandem ones) with the surface of a nanoparticle. In the absence of probes, owing to the proven slow adsorption of double-stranded DNA on AuNPs, the nascent tandem duplexes containing the mutant target will not be able to give rise to aggregates of AuNPs. As a consequence, AuNPs will aggregate under the high-salinity detection conditions. In the case of the wildtype target, the resulting duplexes can adsorb on AuNPs, thereby protecting them from salt-induced aggregation. On the other hand, the observed colorimetric difference was less pronounced as compared with the system containing fluorescent or Lipo-containing anchors, and the reason is a difference in rates of adsorption processes between these two cases.

**Figure 8:**
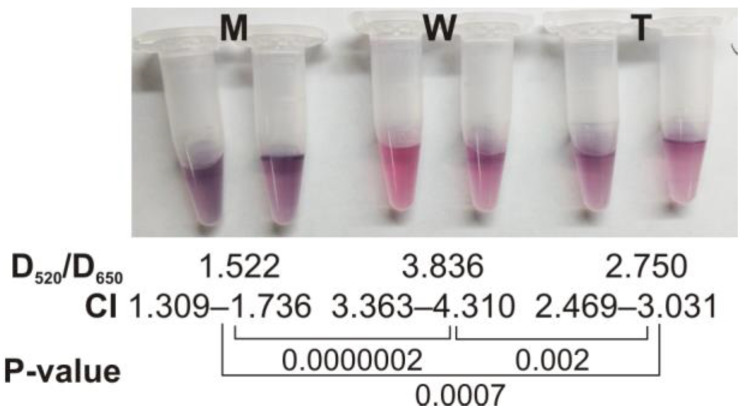
Discrimination between targets differing by one nucleotide (targets M and W) or by the entire sequence (targets M and T) by means of probes carrying Lipo-Trityl/Cy5-anchors. Mean values with the confidence interval (CI) are presented. The data were statistically assessed by one-way ANOVA. Significant differences were found between targets (*p*-value < 0.05).

To prove the proposed scheme of interaction between the targets, probes, and AuNPs, experiments were performed with a completely noncomplementary target called T, which is an oligothymidylate of the same length (Figure 8).

The observed difference in the D_520_/D_65_0 ratio between target M (which is complementary to probes) and the completely noncomplementary target T was detectable and had a magnitude similar to the comparison of target M with the target containing one nucleotide mismatch (W). Thus, the selectivity of the newly developed approach was demonstrated on the M and W targets, differing by only one nucleotide, and, in addition, on a target with an oligothymidylate sequence that was completely noncomplementary to the probes.

Next, the experiments were conducted in the absence of one of the probes (FluPm; Table 3). In this case, as expected, the samples containing either target were red, without a difference in optical density. This is because under these conditions, aggregates of AuNPs cannot form.

The system that we created here enabled discrimination of a target at a 2 nM concentration. This characteristic is not worse than the characteristics of published colorimetric assays involving AuNPs for the detection of drug-resistant MTB-associated SNPs in nucleic acids [1,20].

Thus, in a system limited by components (the absence of one of the probes or the absence of a template complementary to the probes) or by detection conditions (an incubation temperature of the components significantly lower than the T_m_ of expected tandem duplexes), there is no discrimination between targets having a single-nucleotide mismatch. It was demonstrated here that under the conditions proposed by us, formation of tandem duplexes only with a fully complementary target was possible (impossible with a target carrying a single-nucleotide mismatch). These conditions led to a difference in the type and efficiency of adsorption of DNA–DNA structures on AuNPs. Ultimately, this difference manifested itself in a colorimetric dissimilarity between the samples. Our interpretation of the observed phenomena is one of several possible ones but was confirmed by the presented data and control experiments. The transition from model to real samples will introduce many new components into the system (proteins, glucose, lipids, hormones, inorganic compounds, etc.), some of which are capable of interacting with AuNPs and co-adsorbing on their surface or displacing oligonucleotides adsorbed on AuNPs. Moreover, the adsorption of these additional components can certainly affect the colloidal stability of the AuNPs and, accordingly, change the conditions for salt aggregation of samples for the purpose of target discrimination.

## 3. Materials and Methods

### 3.1. Reagents and Chemicals

Tetrachloroauric acid (HAuCl_4_∙4H_2_O) was purchased from Aurat (Moscow, Russia), and sodium citrate dehydrate (Na_3_C_6_H_5_O_7_), ethylenediaminetetraacetic acid (EDTA), 1-bromo-4-(dodecyloxy)benzene, and ethyl benzoate from Sigma–Aldrich (Darmstadt, Germany). Stains-all was purchased from Acros (Princeton, NJ, USA), Tris from Suzhou Yacoo Science (Suzhou, China), H_3_BO_3_ was bought from Soyuzkhimprom (Novosibirsk, Russia), acetonitrile from RCI Labscan (Bangkok, Thailand), Cy5 NHS ester from Lumiprobe (Moscow, Russia), NaCl from Honeywell (Seelze, Germany), DMSO from Serva (Heidelberg, Germany), and C_2_H_5_OH from Kemerovo Pharmaceutical Factory (Kemerovo, Russia). Magnesium chips, iodine, ethyl acetate, 20% ammonium chloride solution, sodium sulfate, toluene, dichloroethane, dimethylformamide, and oxalyl chloride were acquired from Reakhim (Moscow, Russia). Water was purified by means of a Simplicity 185 system (Millipore) and had a resistivity of 18.2 MΩ cm at 25 °C.

### 3.2. Synthesis of AuNPs

AuNPs were prepared using the classic citrate reduction procedure [57]. The size of AuNPs and their dispersity were determined by TEM and DLS using a Zetasizer Nano ZS Plus instrument (Malvern Instruments, Malvern, UK). The concentration of AuNPs was calculated from absorbance at 520 nm using and extinction coefficient of (8.78 ± 0.06) × 10^8^ M^−1^ cm^−1^ [58].

### 3.3. Synthesis of DNAs

Unmodified DNAs and their derivatives containing an amino group or fluoresceine at the 5′- or 3′-end (Table 4) were synthesized on an ASM-800 (Biosset, Novosibirsk, Russia) following the solid-phase phosphoramidite protocol using DNA phosphoramidites from ChemGenes (Wilmington, MA, USA).

**Figure 9:**
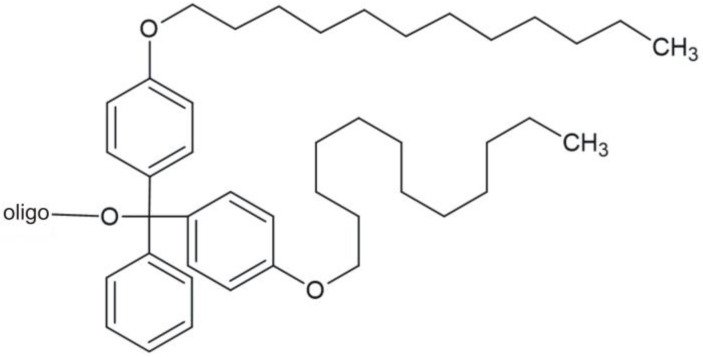
Structure of the Lipo residue in the oligonucleotide probe.

Lipo in the oligonucleotides had the structure shown in Figure 9.

DNAs M, W, and T were purified by electrophoresis in a 10% polyacrylamide gel containing 8 M urea (10% PAAG), and ttgttgPm and ttgttgP were purified by electrophoresis in 15% PAAG. Electrophoresis was carried out in TBE (89 mM Tris, 89 mM H_3_BO_3_, 2 mM EDTA, pH 8.5) for 90 min at 20 V/cm. DNA P-NH_2_ was fluorescently labeled at the 3′-end, using a method from [31], and then was purified by electrophoresis in 15% PAAG containing 8 M urea. DNAs FluPm, FluO, Pm, and P were purified by reversed-phase high-performance liquid chromatography (RP HPLC) on an Agilent 1200 Series using a Zorbax 5 μm Eclipse-XDB-C18 80 Å column (150 × 4.6 mm^2^) from Agilent Technologies (Santa Clara, CA, USA), via a linear gradient of acetonitrile in 0.02 M TEA-Ac (0–50%) during 30 min.

Concentrations of all DNAs were determined using molar coefficients of absorption of the dinucleotides at a wavelength of 260 nm [59].

### 3.4. 4,4’-(Chloro(phenyl)methylene)bis((dodecyloxy)benzene) Synthesis

**Scheme 1:**
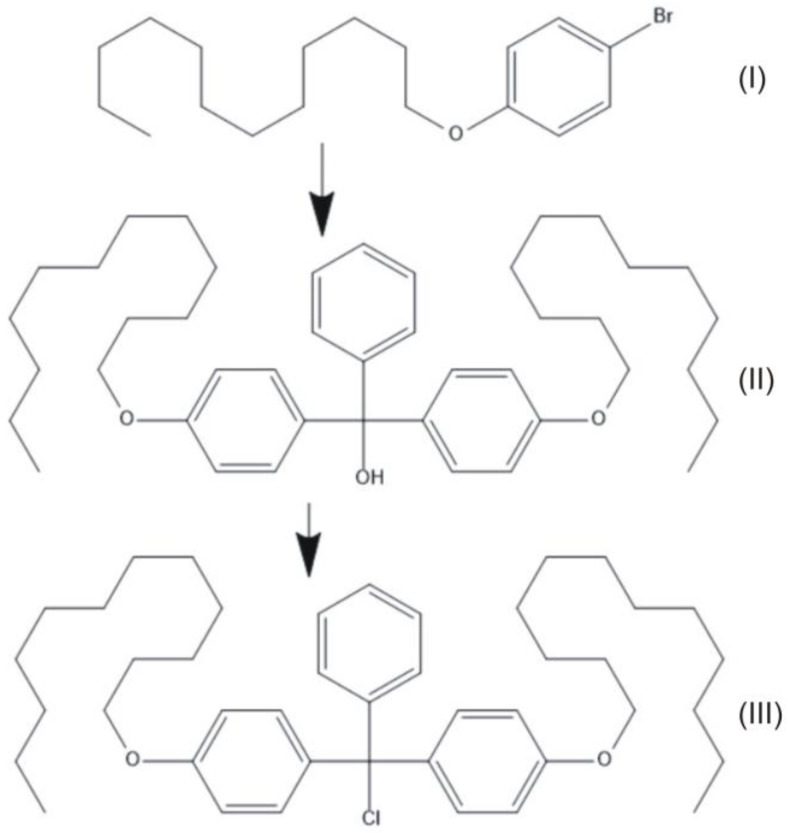
4,4’-(Chloro(phenyl)methylene)bis((dodecyloxy)benzene) synthesis. Structures of the initial compound 1-bromo-4-(dodecyloxy)benzene (**I**), the intermediate product 4,4’-(hydroxy(phenyl)methylene)bis((dodecyloxy)benzene) (**II**), and the final product 4,4’-(chloro(phenyl)methylene)bis((dodecyloxy)benzene) (**III**) are shown.

A solution of 1.18 g (3.46 mmol) of 1-bromo-4-(dodecyloxy)benzene (Scheme 1I) in 5 mL of dry tetrahydrofuran was mixed with 0.15 g of magnesium chips and 2 mg of iodine, placed in an argon atmosphere, ultrasonicated for 10 min, and then heated to 60 °C under reflux conditions. After 1 h, 0.2 mL of ethyl benzoate was added to the mixture and stirred for 6 h. The solution was then quenched with 1 mL of water, mixed with 50 mL of ethyl acetate, washed with 50 mL of 20% ammonium chloride solution, 50 mL of water, and 10 mL of a saturated sodium chloride solution, dried with sodium sulfate, filtered, and evaporated under reduced pressure. The mixture was purified by flash chromatography on a silica gel column in toluene.

The product (Scheme 1II) was dissolved in 1.2 mL of dichloroethane, mixed with 2 drops of dimethylformamide and 0.06 mL of oxalyl chloride, and stirred for 1 h. The product was evaporated under reduced pressure, thus yielding 0.38 g of 4,4’-(chloro(phenyl)methylene)bis((dodecyloxy)benzene) (yield 42%). ^1^H NMR (80 MHz, CDCl3, δ, ppm): 7.52–6.52 (m, 13H), 1.68 (br.s., 4 H), 1.23 (br.s., 40H), 0.83 (br.s., 6H) (Scheme 1III).

### 3.5. CPG-Bound Oligonucleotide Lipophilization

A reactor full (50 μL, 10 mg) of a CPG-bound detritylated oligonucleotide was mixed with 0.3 mL of a 1 M solution of 4,4’-(chloro(phenyl)methylene)bis((dodecyloxy)benzene) in dry pyridine under argon. The mixture was shaken for 3 h, after which it was centrifuged, the solution was decanted, and the solid was washed with 0.5 mL of pyridine twice and 0.5 mL of acetonitrile twice and dried under reduced pressure. Further workup was standard for synthetic oligonucleotides [60]. DNA Lipo-Pm was purified by RP HPLC on an Agilent 1200 Series using a Zorbax 5 μm Eclipse-XDB-C8 80 Å column (150 × 4.6 mm^2^) from Agilent Technologies (Santa Clara, CA, USA), via a linear gradient of acetonitrile in 0.02 M TEA-Ac (0–90%) during 30 min.

### 3.6. Determination of T_m_ Values for DNAs Under Study

Determination of the T_m_ values for all studied DNA duplexes was carried out by the method of thermal denaturation of DNA solutions with optical registration of the signal based on the nearest-neighbor model [61,62,63,64] in (1) 10 × 10^−6^ M DNA solutions in the presence of 5 mM PBS or (2) 0.3 × 10^−6^ M DNA solutions in the presence of 0.3 mM PBS using a CARY 3500 spectrophotometer (Varian, Palo Alto, CA, USA). The measurement error was 0.1 °C. The data were processed using the Simplex software v.2 [65]. For (3) 3 × 10^−9^ M DNA solutions in the presence of 3 mM PBS, the web service OligoCalc v.3.27 was used [38].

### 3.7. SNP Detection Protocol

Firstly, a mix of probes was prepared containing 10 nM Pm and 10 nM P in 0.025× SSC buffer (4 mM NaCl, 0.4 mM Na_3_C_6_H_5_O_7_). A 2.6 nM AuNP solution and solutions containing 15 nM target DNA M, W, or T in water were also prepared and incubated at 52 °C. For sample preparation, aliquots of the probe mixture (102.6 μL) were denatured at 95 °C for 3 min, then a wildtype, mutant, or control target mixture (55.4 μL) was added at 52 °C and incubated for 5 min. The 2.6 nM AuNP solution (98 μL) was added to the samples and incubated at 52 °C for 10 min. Finally, 1x SSC buffer (150 mM NaCl, 15 mM Na_3_C_6_H_5_O_7_, pH 7.4, 28.4 μL) was added to the samples and incubated at 52 °C for 10 min. The procedure was repeated one more time. After that, the samples were subjected to analyses.

### 3.8. UV–Vis Spectroscopy

Optical absorption spectra of the DNA probes with AuNPs were recorded in 200 μL aliquots using a Clariostar plate fluorimeter (BMG Labtech, Ortenberg, Germany) at 520 and 650 nm according to the manufacturer’s instructions.

### 3.9. DLS

Associates of AuNPs with DNA were analyzed using a Malvern Zetasizer Nano (Malvern Instruments, Worcestershire, UK), according to the manufacturer’s instructions, to determine the hydrodynamic diameter of the associates. Each measurement was performed for thirty runs.

### 3.10. RP HPLC of DNA Probes in Analytical Mode

Hydrophobicity of DNAs was assessed as retention time values by RP HPLC Milichrom A-02 (Econova, Novosibirsk, Russia) using the RP column ProntoSil 120-5-C8 (Econova, Novosibirsk, Russia) via gradient elution with acetonitrile (0–90%) in 0.02 M TEA-Ac (pH 6.0) during 15 min at an elution rate of 0.2 mL/min.

### 3.11. TEM

A drop of an AuNP/Cy5P + LipoPm + M or AuNP/Cy5P + LipoPm + W suspension was adsorbed on formvar-coated copper grids for 1 min, and the excess of the liquid was removed with filter paper. The grids were air dried and examined under a JEM 1400 transmission electron microscope (Jeol, Tokyo, Japan) equipped with a Veleta digital camera (EM SIS, Münster, Germany) and the iTEM software, version 5.2 (EM SIS, Münster, Germany).

### 3.12. Statistics

The data obtained by the methods described in Section 3.8, Section 3.9 and Section 3.11 were statistically processed by one-way ANOVA, and differences with a *p*-value < 0.05 were considered statistically significant. The Online ANOVA Calculator [66] was used for this analysis. Data are expressed as means with confidence intervals (CIs) or with standard deviation (±SD) for at least four independent experiments.

## 4. Conclusions

It is known that tuberculosis with extensive drug resistance, including additional drug resistance of MTB to fluoroquinolone, is one of the drug-resistant tuberculoses that are most difficult to treat. Two targets (wildtype and mutant) were chosen here to detect a SNP in a region of the MTB gyrA gene (which is responsible for resistance to fluoroquinolones), as were two probes: one fully complementary to the 5’-terminal region of both targets and the second one fully complementary to the 3’-terminal region of the mutant target but carrying a one-nucleotide mismatch relative to the wildtype target. The probes contained different types of “anchor” residues for efficient adsorption on the surface of AuNPs. Thermal stability of duplexes formed by pairs of targets and probes was assessed. It was found that the difference in melting temperature—between duplexes formed by probe FluPm (specific to the variable part of targets) with target M or with target W—reached 4.2 °C.

The picture of the processes occurring in the system of a target, probes, and AuNPs is a complex phenomenon having distinct thermodynamic, structural, and kinetic features. Accordingly, taking into consideration the pronounced competition between DNA duplex formation and adsorption of individual DNA strands on the AuNP surface [30], a basic scheme for detecting a SNP was devised here that consists of two stages. At the first stage, duplexes formed that were composed of a target and two probes, and this process proceeded with different efficiencies when the two targets (differing by the SNP) were compared. At the second stage, to the pre-formed DNA duplexes, AuNPs were added, which initiated processes of adsorption of the formed duplexes, of free probes, and of targets on the surface of AuNPs. These three processes differed in efficiency, thereby leading to differences in resistance of the final AuNP–DNA associates to salt-induced aggregation. An increase in ionic strength of the solution of a mixture of AuNPs and DNA not only promoted salt-induced aggregation of AuNPs but also stimulated adsorption of the DNA (on the surface of AuNPs): both single-stranded molecules and DNA molecules that formed duplexes of different compositions. This phenomenon reduced the detectable difference between samples containing dissimilar targets. Accordingly, via optical spectroscopy, DLS, and TEM, conditions for duplex formation (with subsequent adsorption of DNA on AuNPs) were determined here that allowed us to differentiate the mutant target from the wildtype target at a 2 nM concentration, owing to the formation of nano-constructs of dissimilar structures. The specificity of this system to the target, which formed a tandem duplex with probes, was demonstrated by means of a noncomplementary target. Although this study was performed on a model system, the achieved limit of detection of the SNP will enable us to proceed to assays of real clinical samples at the next stage of this research. This is a new challenge that researchers have to overcome when developing any new diagnostic platform.

## Figures and Tables

**Table 1:** Calculation of D_520_/D_650_ ratios during discrimination between M (mutant) and W (wildtype) targets by means of probes P and Pm, with various anchors or without them. Mean values with the confidence interval (CI) are presented. The data were statistically assessed by one-way ANOVA. Significant differences were found between targets, with *p*-values < 0.05.

Probes	M		W		
	D_520_/D_650_ Mean	CI	D_520_/D_650_ Mean	CI	*p*-Value
P + Pm	2.708	2.488–2.928	3.245	3.133–3.357	0.001
FluP + FluPm	3.872	3.657–4.087	4.910	4.767–5.053	0.0000009
Cy5P + LipoPm	1.522	1.309–1.736	3.836	3.363–4.310	0.0000001
ttgttgP + ttgttgPm	4.968	4.532–5.404	4.929	4.577–5.281	0.873

**Table 2:** Hydrodynamic diameter (d_H_) and the polydispersity index (PdI) determined by dynamic light scattering (DLS) in samples featuring the didodecyltrityl anchor in probe Pm and the Cy5 residue in probe P and two different targets, M (mutant) or W (wildtype). Mean values with standard deviation (±SD) are presented. The data were statistically assessed by one-way ANOVA. Significant differences between targets were found, with *p*-value = 0.003.

Sample		Mean d_H_, nm (±SD)		PdI (±SD)
AuNP/Cy5P + LipoPm + M		154 ± 80 (99.6%)	4684 ± 789 (0.4%)	0.266 ± 0.012
AuNP/Cy5P + LipoPm + W	10 ± 3 (1%)	79 ± 44 (96.9%)	3684 ± 1074 (2.1%)	0.390 ± 0.015

**Table 3:** Calculation of D_520_/D_650_ ratios under the conditions that do not facilitate discrimination between mutant (M) and wildtype (W) targets by means of probes that carry various anchors or are devoid of them. Mean values with the confidence interval (CI) are presented. The data were statistically assessed by one-way ANOVA. No significant differences were statistically found between targets (*p*-value > 0.05).

Probes	M		W	
	D_520_/D_650_ Mean	CI	D_520_/D_650_ Mean	CI
FluP + FluPm	5.258	5.003–5.513	5.367	5.112–5.622
ttgttgP + ttgttgPm	6.488	5.861–7.115	6.752	6.567–6.937
P + Pm	5.596	5.505–5.687	5.730	5.524–5.936
FluP	3.897	3.605–4.189	4.000	3.729–4.271

**Table 4:** Sequences of single-stranded oligonucleotides (ssDNAs).

Name	Nucleotide Sequence 5′ → 3′
M	5′-CCC-GCA-CGG-CGA-CGC-GTC-GAT-CTA-CGC-CAC-CCT-G-3′
W	5′-CCC-GCA-CGG-CGA-CGC-GTC-GAT-CTA-CGA-CAC-CCT-G-3′
FluPm	5′-FAM-CAG-GGT-GGC-GTA-GAT-CG-3′
FluP	5′-ACG-CGT-CGC-CGT-GCG-GG-FAM-3′
ttgttgPm	5′-TTG-TTG-CAG-GGT-GGC-GTA-GAT-CG-3′
ttgttgP	5′-ACG-CGT-CGC-CGT-GCG-GG-TTGTTG-3′
Pm	5′-CAG-GGT-GGC-GTA-GAT-CG-3′
P	5′-ACG-CGT-CGC-CGT-GCG-GG-3′
LipoPm	5’-Lipo-Tr-CAG-GGT-GGC-GTA-GAT-CG-3’
P-NH2	5’-ACG-CGT-CGC-CGT-GCG-GG-NH2-3’
T	5’-TTT-TTT-TTT-TTT-TTT-TTT-TTT-TTT-TTT-TTT-TTT-T-3’

## Data Availability

Data are available upon request from the corresponding author.

## Supplementary Materials

The following supporting information can be downloaded at: https://www.mdpi.com/article/10.3390/ijms252413230/s1.
