# Supplementary material for: An Approach to Identifying Single-Nucleotide Mutations Using Noncovalent Associates of Gold Nanoparticles with Fluorescently Labeled Oligonucleotides
Source: Int J Mol Sci. 2024 Dec 10;25(24):13230. doi: 10.3390/ijms252413230 (PMC11675405; doi:10.3390/ijms252413230)
Supplement: Supplementary file 1 [file ijms-25-13230-s001.zip › ijms-3273891-supplementary.pdf]

Supplementary material for article "An approach to identifying single-nucleotide mutations using noncovalent associates of gold nanoparticles with fluorescently labeled oligonucleotides"

Table S1. T_m values of duplexes formed by targets M (mutant) and W (wild type) and fluorescein-containing probes P and Pm for the purpose of SNP detection.

Probes	T_m, °C Conditions 1		Conditions 2				Conditions 3[1]	
	M	W	M		W		M	W
FluP	77.3	77.5	-		-		-	-
FluPm	67.1	61.5	58.3		56.7		57.1	54.6
FluP+FluPm	-	-	60.5[I]	69.7[II]	56.3[I]	69.7[II]	-	-
ttgttgP+ttgttgPm	-	-	59.3[I]	67.2[II]	54.0[I]	66.6[II]	-	-

[1] OligoCalc calculates Tm of duplexes without taking into account overhangs within them; therefore, strictly speaking, the obtained values are applicable to blunt-ended duplexes. Conditions 3 are described below.

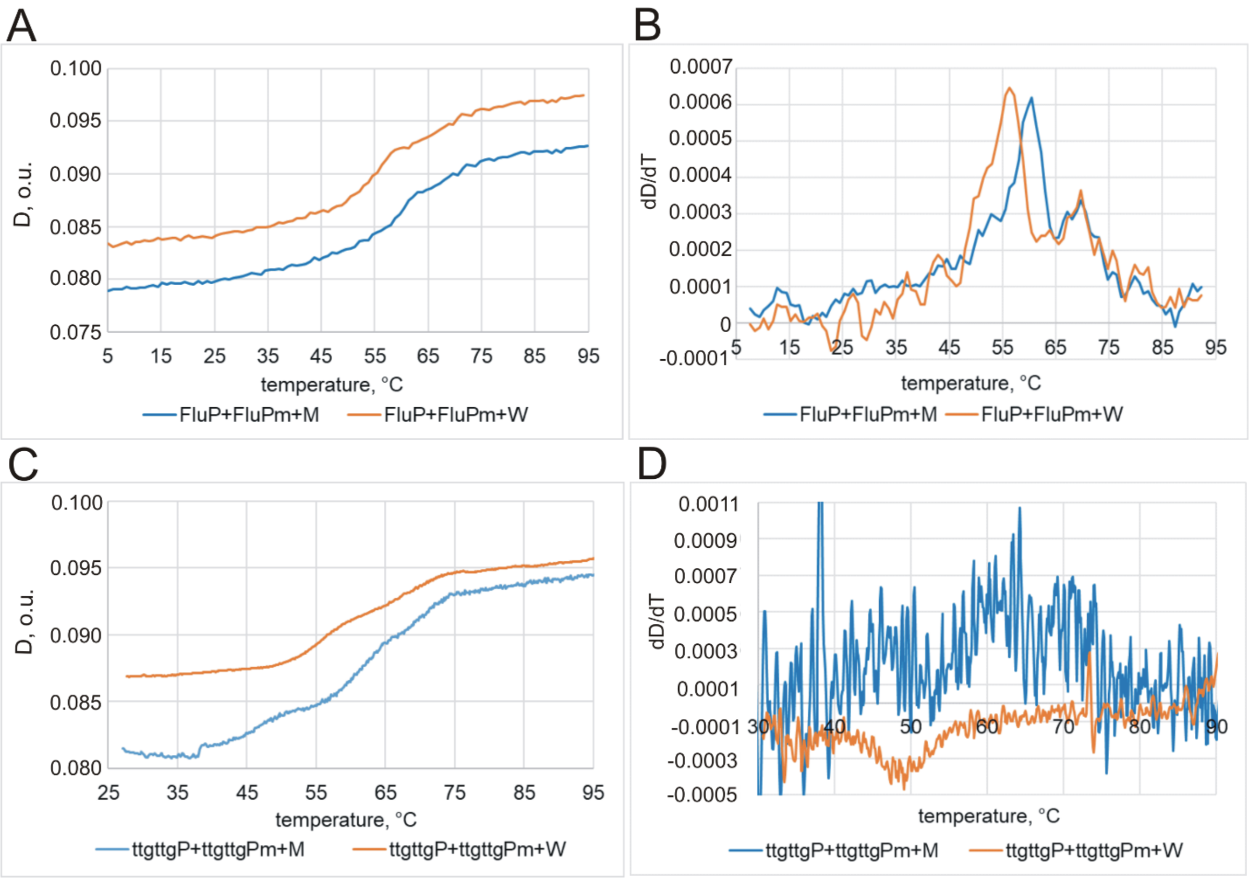

Figure S1. Thermal-denaturation integral and differential curves of tandem duplexes formed by targets M (mutant) and W (wild type) and Flu-containing probes P and Pm (A, B) or ttgttg-containing probes P and Pm (C, D).
